# Follow up: Advancing the activity cliff concept, part II

**DOI:** 10.12688/f1000research.3788.1

**Published:** 2014-03-18

**Authors:** Dagmar Stumpfe, Antonio de la Vega de León, Dilyana Dimova, Jürgen Bajorath

**Affiliations:** 1 Department of Life Science Informatics, B-IT, LIMES Program Unit Chemical Biology and Medicinal Chemistry, Rheinische Friedrich-Wilhelms-Universität, Bonn, D-53113, Germany

## Abstract

We present a follow up contribution to further complement a previous commentary on the activity cliff concept and recent advances in activity cliff research. Activity cliffs have originally been defined as pairs of structurally similar compounds that display a large difference in potency against a given target. For medicinal chemistry, activity cliffs are of high interest because structure-activity relationship (SAR) determinants can often be deduced from them. Herein, we present up-to-date results of systematic analyses of the *ligand efficiency* and *lipophilic efficiency* relationships between activity cliff-forming compounds, which further increase their attractiveness for the practice of medicinal chemistry. In addition, we summarize the results of a new analysis of *coordinated activity* *cliffs* and clusters they form. Taken together, these findings considerably add to our evaluation and current understanding of the activity cliff concept. The results should be viewed in light of the previous commentary article.

## Introduction

Over the past decade, the activity cliff concept has been increasingly discussed in the chemoinformatics and medicinal chemistry literature ^1– 3^. In the practice of medicinal chemistry, activity cliffs, which are formed by structurally similar or analogous compounds with large potency differences for a given target ^1, 2^, have long been considered during chemical optimization efforts, typically for individual compound series ^2^. However, the increasing popularity of the activity cliff concept can at least in part be attributed to computational exploration and large-scale analysis ^2, 3^. In fact, much of our current knowledge about activity cliffs has resulted from compound data mining and other chemoinformatics investigations ^2– 4^. Hence, in addition to supporting practical applications in compound development, activity cliff research is an area where chemoinformatics and medicinal chemistry meet.

In a previous commentary ^4^, we have summarized key aspects of the activity cliff concept and discussed further extensions and refinements. Among others, discussed topics have included the current frequency of occurrence of activity cliffs, their dependence on chosen molecular representations, their target distributions, and associated structure-activity relationship (SAR) information ^4^. Herein, we present a follow up to this commentary, which has been catalyzed by the availability of new results concerning the ligand efficiency and lipophilic efficiency of activity cliff partners as well as the topology of coordinated activity cliffs formed across currently available bioactive compounds. These findings should also be considered as further advancements of the activity cliff concept and viewed on the basis of the previous commentary article.

## Activity cliff definition

The definition of activity cliffs requires the specification of a *similarity criterion* (when are two compounds “similar”?) and a *potency difference criterion* (when is a potency difference “large” and “significant”?) ^1, 2^. Molecular similarity can be assessed in a variety of ways, which can roughly be divided into chemical descriptor-based approaches, which require the calculation of similarity values based on the comparison of chosen molecular representations ^2^, and substructure-based approaches, which directly establish structural relationships (on the basis of molecular graphs) ^2^. Among substructure-based approaches, the matched molecular pair (MMP) formalism ^5^ has become very popular in recent years. An MMP is defined as a pair of compounds that are distinguished by the exchange of a substructure at a single site ^5^ termed a chemical transformation ^6^. The formation of an MMP can thus be considered as a possible similarity criterion for activity cliff formation. To define activity cliffs, transformation size-restricted MMPs have been introduced in which transformations are limited to small and chemically meaningful replacements ^7^. Accordingly, transformation size-restricted MMPs mostly account for structural analogs.

In the following, we consistently adhere to our previously rationalized preferred activity cliff definition ^4^:

*(a) Similarity criterion: Formation of a transformation size-restricted MMP.*

*(b) Potency difference criterion: At least two orders of magnitude (≥ 100-fold).*

*(c) Activity measurements: Equilibrium constants (K _i_ values).*

So defined activity cliffs have also been termed MMP-cliffs ^7^.

## Ligand efficiency and lipophilic efficiency

For the assessment of activity cliffs, compound potency has thus far been a focal point, consistent with the original activity cliff definition. However, during compound optimization, other criteria are often applied to monitor progress that relate potency to changes in molecular weight (MW) or hydrophobicity ^8^. These criteria are formalized as optimization indices and include, among others, *ligand efficiency* (LE) ^8, 9^ or *ligand lipophilic efficiency* (LLE) ^8, 10^, which is also termed *lipophilic efficiency* (LipE) ^8, 11, 12^. For an active compound, LE yields the fraction of potency per non-hydrogen atom or MW unit ^9^. In the presence of strong and specific ligand-target interactions, LE should increase during compound optimization; in other words, a gain in potency should not primarily be attributed to molecular size effects. Herein, LE was calculated using the binding efficiency index (BEI) ^13^ defined as:

             BEI (LE) = pK _i_/MW [log unit/kDa]

Furthermore, LipE was calculated as ^10^:

             LipE (LLE) = pK _i_ – cLogP [log unit].

LipE is obtained by subtracting the logarithm of the calculated octanol/water partition coefficient, a measure of hydrophobicity, from the logarithm of the equilibrium constant. Hence, LipE indirectly accounts for the influence of hydrophobicity on potency. LipE should also increase during compound optimization because a gain in potency should not primarily be attributed to increasing hydrophobicity of a compound (which often gives rise to non-specific binding effects).

Because LE and LipE are important and widely applied measures for compound optimization in drug discovery, it makes sense to also consider them in the context of activity cliff formation, given their immediate relevance for SAR analysis. In a recent analysis ^14^, 18,208 activity cliffs were extracted from more than 41,000 unique ChEMBL ^15^ (release 15) compounds with activity against the current spectrum of human targets. Then, differences in LE between lowly and highly potent cliff partners were systematically assessed. For activity cliffs based upon our preferred definition, one would hope that favorable changes in LE might be observed in many instances. However, whether or not systematic trends might be detectable was an open question. The analysis revealed that the formation of 99.1% of all activity cliffs across different targets was accompanied by consistent increases in LE values between lowly and highly potent cliff partners, with, on average, ∆LE = 6.25 ^14^. For activity cliffs defined on the basis of calculated (molecular fingerprint-based) similarity values, comparable observations were made ^14^.

**Figure 1.**
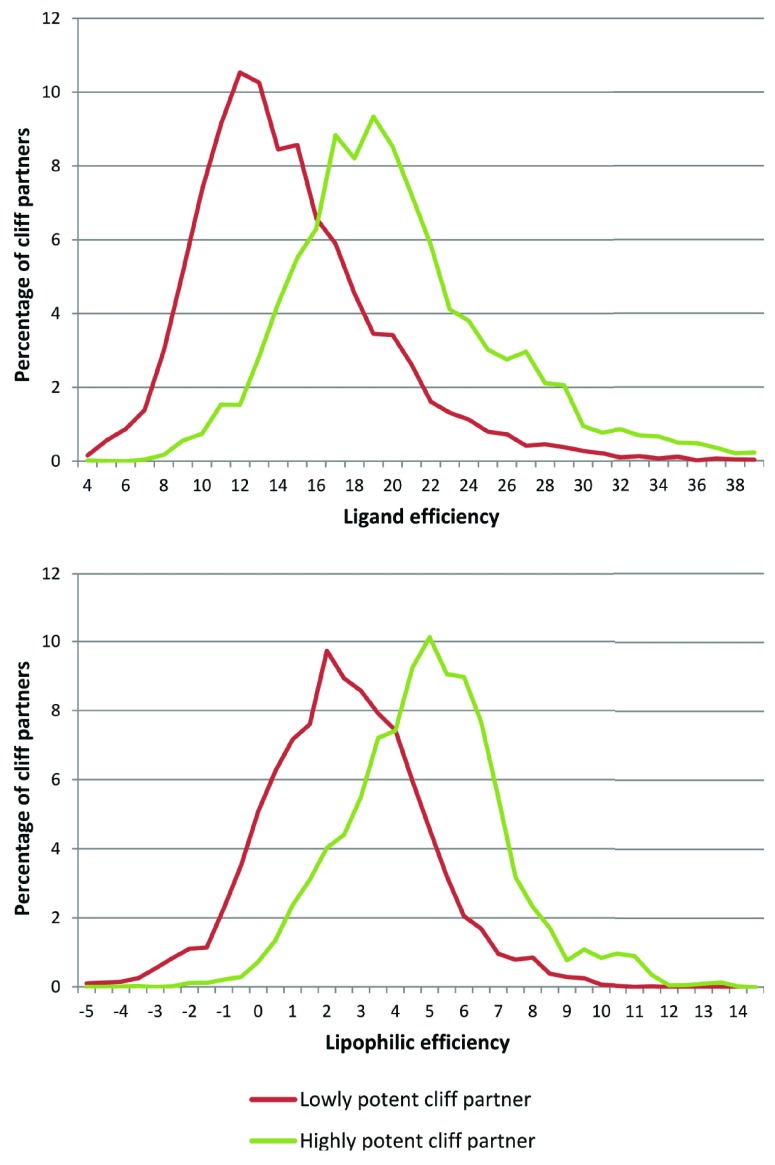
Ligand and lipophilic efficiency value distributions. LE (top) and LipE (bottom) value distributions are shown for lowly (red line) and highly potent (green) cliff partners.

Here, we report results of LE and, in addition, LipE analysis for the most recent release of the ChEMBL database (version 17). From a total of ~45,000 unique ChEMBL compounds active against 661 different human targets (~77,000 K _i_ values), 20,080 activity cliffs were isolated. For the highly and lowly potent partners of each cliff, LE and LipE were calculated. The resulting value distributions are displayed in Figure 1. An increase in LE and LipE values for the highly potent cliff partner was detected for 99.1% and 96.7% of all activity cliffs, respectively, with, on average, ∆LE = 6.27 and ∆LipE = 2.42. Hence, similarly positive LE and LipE trends were observed for activity cliff formation (for LipE, this was difficult to predict). These findings further emphasize the relevance of activity cliff information for medicinal chemistry applications because chemical modifications encoded by activity cliffs consistently increase potency, LE, and LipE.

## Coordinated activity cliffs

The assessment of activity cliffs has conventionally focused on compound pairs. Hence, cliffs are typically considered on an individual basis (including statistical analysis). However, activity cliffs are only formed in isolation if participating compounds have no structural neighbors with which they also form cliffs. This is unlikely for many compound series and data sets, especially those resulting from compound optimization efforts. In earlier studies, series of highly and lowly potent analogs have been identified in different data sets that formed multiple overlapping activity cliffs ^16^. These cliff arrangements have been termed *coordinated activity cliffs* ^17^. Indeed, based upon global statistical analysis, we have determined that ~97% of all activity cliffs are formed in a coordinated manner ^4^. In principle, coordinated activity cliffs have higher SAR information content than and are thus of particular interest for medicinal chemistry. However, only very little information has thus far been available about how coordinated activity cliffs are formed and what the size of coordinated cliff arrangements might be.

**Figure 2.**
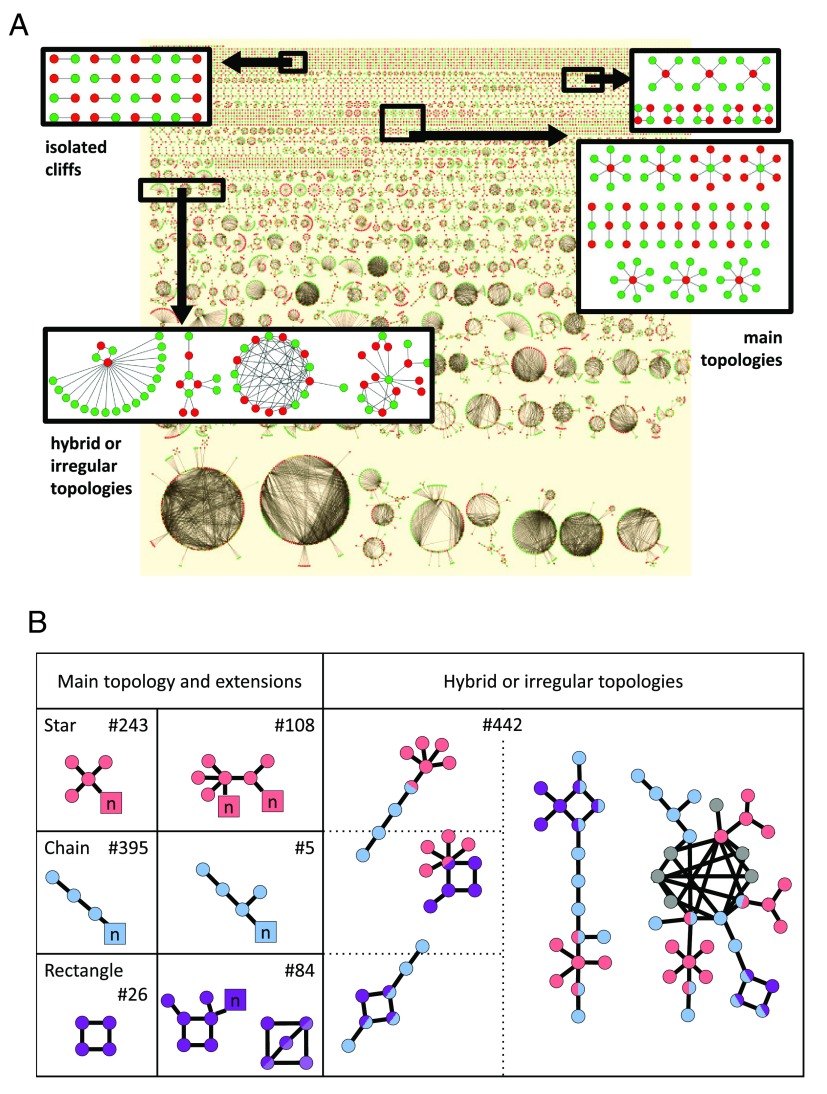
Activity cliff network and cluster topologies. In ( **A**), the complete activity cliff network is shown. Nodes represent compounds and edges activity cliffs. Nodes of highly and lowly potent cliff partners are colored green and red, respectively, and nodes representing a compound that is a highly and lowly potent partner in different activity cliffs are colored yellow. Small sections of the network containing exemplary activity cliff cluster topologies are magnified. On the right, examples of the three most frequently occurring (main) topologies are displayed, which include so-called *star*, *chain*, and *rectangle* topology. In ( **B**), main activity cliff cluster topologies and observed extensions as well as hybrid and irregular topologies are schematically illustrated (pink nodes: *star*; light blue: *chain*; purple: *rectangle* topology; gray: no topology assignment). Dual-color nodes indicate compounds belonging to cluster components with hybrid topologies. Squared nodes represent variable compound numbers ( *n*) for a given topology. For each topology, the number ( *#*) of instances in the network is reported.

Therefore, in a recent study, all activity cliffs extracted from active compounds in ChEMBL (version 17) were subjected to network analysis ^18^. Activity cliff forming compounds were represented as nodes that were connected by edges accounting for individual activity cliffs. The global network is depicted in Figure 2A. It consisted of activity cliffs formed by compounds with activity against a total of 293 targets, and more than 93% of all activity cliffs were found to be single-target cliffs ^18^. Only 769 (3.8%) of a total of 20,080 activity cliffs were formed in isolation. Coordinated activity cliffs appeared as different-sized disjoint clusters of varying topologies. In total, 19,311 coordinated activity cliffs formed 1303 separate clusters. Among these were 26 clusters consisting of more than 50 compounds and 420 clusters containing six to 15 compounds, hence reflecting a high degree of activity cliff coordination.

The activity cliff clusters displayed 449 distinct topologies with different frequency of occurrence. Examples are provided in Figure 2A and 2B. The majority of activity cliff clusters, i.e., 861, were assigned to only three recurrent main topologies, termed the *star*, *chain*, and *rectangle* topology, and a limited number of extensions and combinations of these topologies, as illustrated in Figure 2B. The recurrent topologies covered many clusters of small to medium size. Topologies of increasingly large size often had hybrid character or became irregular, as also illustrated in Figure 2B.

The star topology reflects the presence of a highly or lowly potent compound and multiple analogs having opposite potency, a situation frequently observed in compound optimization. In total, the star topologies and its extensions were detected 351 times. Different from clusters with star topology, chains with more than three compounds and rectangles require the presence of alternating highly and lowly potent compounds forming sequences of activity cliffs or circular arrangements, which are less likely than stars. However, these topologies were also recurrent.

Activity cliff network analysis has revealed how coordinated activity cliffs are formed across currently available compound activity classes. Taken together, the results clearly indicate that many coordinated activity cliffs occur as well-defined clusters with recurrent topologies, which can be easily isolated and subjected to SAR exploration. For a detailed characterization of the global activity cliff network and individual cluster topologies, the interested reader is referred to the original publication ^18^.

## Conclusions

Herein, we have presented an update on the state-of-the-art in rationalizing activity cliffs. Focal points of our analysis have been the large-scale characterization of activity cliffs in terms of ligand efficiency and lipophilic efficiency as well as the visualization and systematic assessment of coordinated activity cliffs. The finding that activity cliff formation is generally accompanied by improvements in ligand and lipophilic efficiency further increases the attractiveness of activity cliff information for compound optimization. In addition, the observation that coordinated activity cliffs often form clusters of well-defined topology, irrespective of specific compound activities, is relevant for SAR analysis. Because activity clusters are rich in SAR information, an important topic for future research will be how such SAR information might be systematically extracted from clusters with different topology.
