# Supplementary material for: Follow up: Advancing the activity cliff concept, part II
Source: F1000Res. 2014 Mar 18;3:75. [Version 1] doi: 10.12688/f1000research.3788.1 (PMC3983935; doi:10.12688/f1000research.3788.1)
Supplement: Peer review history

# Peer review: Referee response for version 1

**DOI:** 10.5256/f1000research.4057.r4182

**Published:** 2014-04-02

**Authors:** J. Richard Morphy

**Affiliations:** 1 Department of Medicinal Chemistry, Lilly Research Laboratories, Surrey, UK

The twin concepts of molecular matched pair (MMP) analysis and activity cliffs have been widely embraced by medicinal chemists in recent years as a means to explore and understand complex SAR landscapes. The contributions of the Bajorath group have been pivotal to the acceptance of these approaches. Activity cliffs are large changes in a biological activity arising from modest structural differences. In this study size restrictions are imposed on the activity cliff/MMP partners to ensure that structural changes are relatively small. Therefore it might be expected that the activity cliff trends based upon ligand efficiency would mirror those from activity alone. Still it is reassuring that this paper confirms that activity cliffs are indeed associated with large benefits in terms of ligand efficiency. The more interesting observation is that activity cliffs are also associated with significant improvements in lipophilic ligand efficiency (LLE or LipE) since this is less intuitive based on the selection criteria used for the MMP analysis. Therefore this latest study adds real weight to the argument that MMPs and activity cliffs can be very useful concepts within hit and lead optimisation projects.

I have read this submission. I believe that I have an appropriate level of expertise to confirm that it is of an acceptable scientific standard.

# Peer review: Referee response for version 1

**DOI:** 10.5256/f1000research.4057.r4181

**Published:** 2014-03-21

**Authors:** Gerhard Müller

**Affiliations:** 1 Medicinal Chemistry, Mercachem, Nijmegen, Netherlands

Dagmar Stumpfe, Antonio de la Vega de Leon, Dilyana Dimova, and Jürgen Bajorath refer in the submitted commentary entitled “ ***Follow up: Advancing the activity cliff concept, part II***” to a previously published commentary on the activity cliff concept, now reporting on (i) how this concept correlates with ligand efficiency measures of “cliff-forming” compound pairs, and (ii) how "coordinated activity cliffs" give new insight into a putative course of optimization by tracing the activity-critical compounds introducing activity cliff network and cluster topologies.

The group around Jürgen Bajorath has made major contributions over the last few years to the development and establishment of the activity cliff concept within Medicinal Chemistry, documented by numerous seminal publications in the field, that provide a constantly maturing cheminformatics tool useful for the medicinal chemist to assess the steepness of an unfolding structure-activity relationship landscape. Activity cliff-forming compounds give rise to sharp curvatures in the structure-activity relationship surface and thus might suggest that affected groups establish significant pharmacophoric features in an underlying compound class. It is especially the introduction and consequent application of so-called “transformation size-restricted matched molecular pairs” that renders this concept useful for the end-user, since an abstract cheminformatics tool becomes more tangible for the practitioner in the field.

In this context the authors set out to consider as to whether the occurrence of activity cliffs is associated with a general and systematic increase in molecular weight, as this is often found in consecutive medicinal chemistry optimization rounds (e.g. transforming a primary hit into a structurally more elaborate lead-like compound).This optimization process is often accompanied by a significant increase in molecular weight, as well as in lipophilicity. For that reason, normalization of detected binding affinities or inhibition data onto e.g. the number of heavy atoms in an underlying molecule, allows to better assess and cross-compare the true efficiency of a given compound acting at a specific target. Compounds of high ligand efficiency but low molecular weight and low lipophilicity are preferred in that they show fewer liabilities in e.g. unspecific binding to other targets, or in the number of putative metabolic soft spots.

By applying stringent quality criteria for data mining, the authors have carried out a systematic analysis within the ChEMBL version 17 database identifying MMP cliffs. Approximately 20,000 activity cliffs were generated and systematically analysed for ligand efficiency changes. In more than 99% of all activity cliffs, the more potent cliff-forming compound exhibits consistently higher ligand efficiency, and in more than 96% of all identified MMP cliffs, the more active compound showed an increased lipophilic efficiency. And this finding is derived from more than 45,000 distinct compounds with biological activities reported for more than 661 targets, rendering the underlying dataset truly diverse in its chemical and biological nature.

Activity cliff analyses by their algorithmic nature focus on compound pairs, in this contribution so-called matched molecular pairs with a size-restricted transformation accounting for a single structural difference between the two cliff-forming compounds. It seems obvious that the identified and isolated 20,000 MMP-cliffs might cluster into common underlying optimization programs, since the majority of medicinal chemistry optimization campaigns cover a number of subsequent iterative feedback cycles with many structurally related compounds belonging to consecutive design generations. To account for this interdependence of isolated cliff-forming compound pairs, the group around Jürgen Bajorath has embarked into the concept of “coordinated activity cliffs”.

Again, this is a very helpful attempt to back-translate an abstract and simplified view on a compound-activity space into the operational world of medicinal chemistry in which those formerly isolated pairs appear in a broader context of more comprehensive chemistry campaigns. By generating and analysing activity cliff network and cluster topologies, a hypothetical optimization pathway can be unfolded that provides additional guidelines to the practitioner on the optimization philosophy.

Admittedly, the naive end-user being confronted for the first time with the activity cliff concept will require some time to fully appreciate the intrinsic value of this cheminformatics-based approach towards the analysis of structure-activity relationships. However, the concept becomes more and more intuitive and as such is a true asset that should be applied in small-molecule drug discovery projects.

As in the previous commentary of the Bajorath group, I see the attempt to reach high user-friendliness (e.g. by explaining the concept of coordinated activity cliffs) which renders this commentary helpful in alerting the medicinal chemistry community to this very useful, but still under-appreciated concept. The group around Jürgen Bajorath continue to qualify as advocates in that sense, and the community of practicing medicinal chemists should start to move in their direction accordingly.

I have read this submission. I believe that I have an appropriate level of expertise to confirm that it is of an acceptable scientific standard.
